# (*E*)-1-(4-Nitro­benzyl­idene)-2,2-diphenyl­hydrazine

**DOI:** 10.1107/S1600536812043681

**Published:** 2012-10-31

**Authors:** Angel Mendoza, Ruth Meléndrez-Luevano, Blanca M. Cabrera-Vivas, Claudia Acoltzi-X, Marcos Flores-Alamo

**Affiliations:** a Centro de Química, Instituto de Ciencias, Benemérita Universidad Autónoma de Puebla, 72570, Puebla, Pue., Mexico; b Facultad de Ciencias Químicas, Benemérita Universidad Autónoma de Puebla, 72570, Puebla, Pue., Mexico; c Facultad de Química, Universidad Nacional Autónoma de México, 04510, México D.F., Mexico

## Abstract

The asymmetric unit of the title compound, C_19_H_15_N_3_O_2_, contains two mol­ecules, both of which show an *E* conformation of the imine bond. The dihedral angles between the phenyl rings in the phenyl­hydrazine groups are 86.09 (6) and 83.41 (5)° in the two mol­ecules. The 4-nitrobenzene rings show torsion angles of 4.4 (2) and 10.9 (2)° from the two C=N—N planes. In the crystal, C—H⋯π inter­actions and C—H⋯O hydrogen bonds are observed growing along the *a*, *b* and *c* axes, resulting in a complex supramolecular array.

## Related literature

For applications of hydrazones, see: Angell *et al.* (2006 ▶); Vicini *et al.* (2002 ▶); Rollas *et al.* (2002 ▶). 
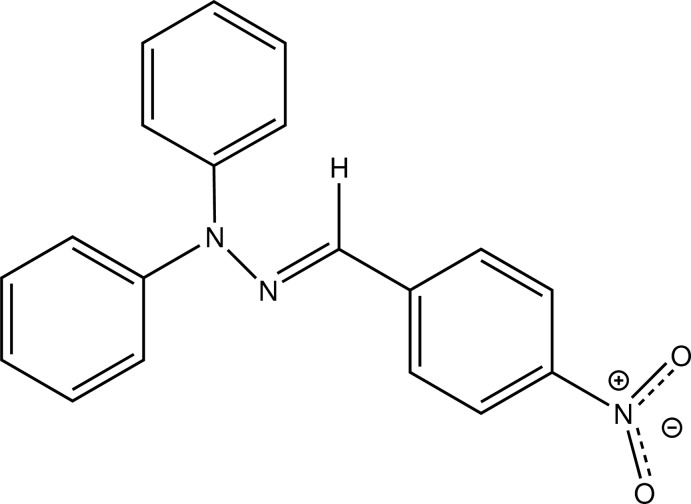


## Experimental

### 

#### Crystal data

C_19_H_15_N_3_O_2_
*M* *_r_* = 317.34
Triclinic, 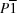

*a* = 10.8648 (6) Å
*b* = 11.1477 (6) Å
*c* = 16.2075 (7) Å
α = 72.084 (4)°
β = 89.037 (4)°
γ = 62.084 (6)°
*V* = 1631.47 (18) Å^3^
*Z* = 4
Mo *K*α radiation
μ = 0.09 mm^−1^
*T* = 298 K
0.6 × 0.36 × 0.29 mm

#### Data collection

Oxford Diffraction Xcalibur (Atlas, Gemini) diffractometer
Absorption correction: analytical (*CrysAlis PRO*; Oxford Diffraction, 2009 ▶) *T*_min_ = 0.963, *T*_max_ = 0.98
11850 measured reflections
6432 independent reflections
3566 reflections with *I* > 2σ(*I*)
*R*_int_ = 0.020

#### Refinement

*R*[*F* ^2^ > 2σ(*F* ^2^)] = 0.038
*wR*(*F* ^2^) = 0.100
*S* = 0.89
6432 reflections
434 parameters
H-atom parameters constrained
Δρ_max_ = 0.19 e Å^−3^
Δρ_min_ = −0.17 e Å^−3^

### 

Data collection: *CrysAlis PRO* (Oxford Diffraction, 2009 ▶); cell refinement: *CrysAlis PRO*; data reduction: *CrysAlis PRO*; program(s) used to solve structure: *SHELXS97* (Sheldrick, 2008 ▶); program(s) used to refine structure: *SHELXL97* (Sheldrick, 2008 ▶); molecular graphics: *ORTEP-3 for Windows* (Farrugia, 1997 ▶); software used to prepare material for publication: *WinGX* (Farrugia, 1999 ▶).

## Supplementary Material

Crystal structure: contains datablock(s) global, I. DOI: 10.1107/S1600536812043681/bt6850sup1.cif

Structure factors: contains datablock(s) I. DOI: 10.1107/S1600536812043681/bt6850Isup2.hkl

Supplementary material file. DOI: 10.1107/S1600536812043681/bt6850Isup3.cml

Additional supplementary materials:  crystallographic information; 3D view; checkCIF report

## Figures and Tables

**Table 1:** Hydrogen-bond geometry (Å, °) *Cg*1, *Cg*2 and *Cg*3 are the centroids of the C21–C26, C33–C38 and C8–C13 rings, respectively.

*D*—H⋯*A*	*D*—H	H⋯*A*	*D*⋯*A*	*D*—H⋯*A*
C3—H3⋯*Cg*1^i^	0.93	2.92	3.4080 (18)	114
C29—H29⋯*Cg*2^ii^	0.93	2.80	3.6875 (18)	161
C7—H7⋯*Cg*2	0.93	2.83	3.4223 (16)	123
C30—H30⋯*Cg*3^iii^	0.93	2.84	3.698 (2)	154
C6—H6⋯O2^iv^	0.93	2.60	3.342 (3)	138

Symmetry codes: (i) 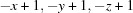; (ii) 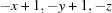; (iii) 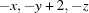; (iv) 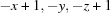.

**Fig. 1.**
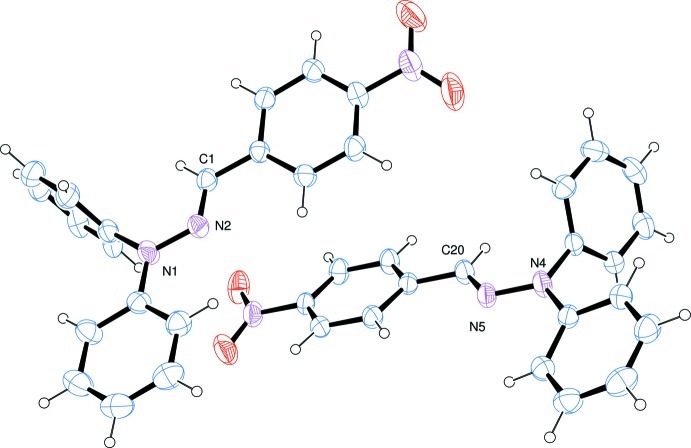
The molecular structure of title compound, with atom labels and 30% probability displacement ellipsoids for non-H atoms.

## supplementary crystallographic information

### Comment

Hydrazones have had diverse applications in pharmacology, microbiology and the industry; some of them are used in analytical tests, which serve to detect chemical and biological species (Angell, *et al.*, 2006). Some hydrazones with functional groups like NO_2_ and Cl, have been studied to have potential antimicrobial agents and were tested for their antibacterial and antifungal activities against (Vicini, *et al.*, 2002 and Rollas *et al.*, 2002). In the industry, hydrazones are used as plasticizing agents, polymerization initiators and antioxidants.

In the title compound C_19_H_15_N_3_O_2_, the ASU contains two molecules showing an *E* configuration on each of the C=N groups with diphenylhydrazine group opposite to *p*-nitrophenyl ring. The dihedral angle for phenyl rings C8—C13 and C14—C19 is 86.09 (6)° for molecule 1 and that between C27—C32 and C33—C38 rings is 83.41 (5)° for molecule 2. The dihedral angle for *p*-nitrophenyl rings and C=N—N planes are 10.89 (20) and 4.43 (23)° for molecule 1 and 2 respectively. The imine bond distances [N2—C1 1.2847 (18) Å and N5—C20 1.2774 (17) Å] are typical C=N bond. The crystal packing present four intermolecular interactions of the type C—H···π (table 1). Moreover, there is one intermolecular interaction of type hydrogen bond: C6—H6···O2, and an intramolecular interaction of type hydrogen bond, C15—H15···N2.

### Experimental

Diphenylhydrazine was dissolved in ethanol (1.2 chemical equivalents), a chemical equivalent of aldehyde which was previously dissolved in the same solvent and it was added drop by drop stirring constantly. The reaction mixture was kept at room temperature and was monitored by TLC, and then vacuum filtered. The hydrazones were recrystallized by a continuous and controlled process until orange crystals with adequate size and purity were developed in order to obtain X-ray studies. Yield 90%. UV λ_max_ = 411.51 nm. FT IR (film): (cm^-1^): 3031 ν(C—H), 1591, 1556 ν(C=N), 1508 ν(Ph—NO_2_). ^1^H NMR (400 MHz, (CD_3_)_2_CO: (d/p.p.m.): 8.20–8.18 (m, 2H), 7.88–7.86 (m, 2H), 7.51–7.47 (m, 4H), 7.29–7.22 (m, 7H). ^13^C NMR (400 MHz, (CD_3_)_2_CO): (d/p.p.m.): 143.02, 143.01, 142.80, 132.30, 130.03, 126.58, 125.35, 123.88, 122.43. MS—EI: m/*z* = 317 *M*^+^ C_19_H_15_N_3_O_2_.

### Refinement

H atoms bonded to C atoms were placed in geometrical idealized positions and were refined as riding on their parent atoms, with C—H = 0.93–0.98 Å and with *U*_iso_(H) = 1.2 *U*_eq_(C).

### Crystal data

**Table:**

C_19_H_15_N_3_O_2_	*Z* = 4
*M**_r_* = 317.34	*F*(000) = 664
Triclinic, *P*1	*D*_x_ = 1.292 Mg m^−^^3^
*a* = 10.8648 (6) Å	Mo *K*α radiation, λ = 0.71073 Å
*b* = 11.1477 (6) Å	Cell parameters from 4346 reflections
*c* = 16.2075 (7) Å	θ = 3.6–26.0°
α = 72.084 (4)°	µ = 0.09 mm^−^^1^
β = 89.037 (4)°	*T* = 298 K
γ = 62.084 (6)°	Prism, yellow
*V* = 1631.47 (18) Å^3^	0.6 × 0.36 × 0.29 mm

### Data collection

**Table:**

Oxford Diffraction Xcalibur (Atlas, Gemini) diffractometer	6432 independent reflections
Graphite monochromator	3566 reflections with *I* > 2σ(*I*)
Detector resolution: 10.4685 pixels mm^-1^	*R*_int_ = 0.020
ω scans	θ_max_ = 26.1°, θ_min_ = 3.6°
Absorption correction: analytical (*CrysAlis PRO*; Oxford Diffraction, 2009)	*h* = −13→12
*T*_min_ = 0.963, *T*_max_ = 0.98	*k* = −13→11
11850 measured reflections	*l* = −20→19

### Refinement

**Table:**

Refinement on *F*^2^	Secondary atom site location: difference Fourier map
Least-squares matrix: full	Hydrogen site location: inferred from neighbouring sites
*R*[*F*^2^ > 2σ(*F*^2^)] = 0.038	H-atom parameters constrained
*wR*(*F*^2^) = 0.100	*w* = 1/[σ^2^(*F*_o_^2^) + (0.0527*P*)^2^] where *P* = (*F*_o_^2^ + 2*F*_c_^2^)/3
*S* = 0.89	(Δ/σ)_max_ = 0.001
6432 reflections	Δρ_max_ = 0.19 e Å^−^^3^
434 parameters	Δρ_min_ = −0.17 e Å^−^^3^
0 restraints	Extinction correction: *SHELXL97* (Sheldrick, 2008)
Primary atom site location: structure-invariant direct methods	Extinction coefficient: 0.0160 (12)

### Special details

**Table:**

Geometry. All s.u.'s (except the s.u. in the dihedral angle between two l.s. planes) are estimated using the full covariance matrix. The cell s.u.'s are taken into account individually in the estimation of s.u.'s in distances, angles and torsion angles; correlations between s.u.'s in cell parameters are only used when they are defined by crystal symmetry. An approximate (isotropic) treatment of cell s.u.'s is used for estimating s.u.'s involving l.s. planes.
Refinement. Refinement of *F*^2^ against ALL reflections. The weighted *R*-factor *wR* and goodness of fit *S* are based on *F*^2^, conventional *R*-factors *R* are based on *F*, with *F* set to zero for negative *F*^2^. The threshold expression of *F*^2^ > 2σ(*F*^2^) is used only for calculating *R*-factors(gt) *etc*. and is not relevant to the choice of reflections for refinement. *R*-factors based on *F*^2^ are statistically about twice as large as those based on *F*, and *R*- factors based on ALL data will be even larger.

### Fractional atomic coordinates and isotropic or equivalent isotropic displacement parameters (Å^2^)

**Table:**

	*x*	*y*	*z*	*U*_iso_*/*U*_eq_	
N5	0.37912 (13)	0.62485 (13)	0.71748 (7)	0.0518 (3)	
N4	0.48398 (13)	0.55837 (13)	0.78710 (7)	0.0570 (3)	
N2	0.10818 (12)	0.73321 (14)	0.28854 (8)	0.0526 (3)	
N1	0.00738 (13)	0.84107 (14)	0.22028 (8)	0.0583 (3)	
N6	−0.13482 (15)	0.81987 (18)	0.42567 (8)	0.0646 (4)	
C1	0.14313 (15)	0.60094 (17)	0.30146 (9)	0.0516 (4)	
H1	0.0981	0.5785	0.2651	0.062*	
C21	0.20067 (15)	0.62924 (15)	0.63192 (8)	0.0476 (4)	
C2	0.25354 (14)	0.48631 (16)	0.37320 (9)	0.0468 (4)	
C20	0.31425 (15)	0.56008 (16)	0.70491 (9)	0.0533 (4)	
H20	0.34	0.4676	0.7429	0.064*	
C27	0.53186 (15)	0.41316 (15)	0.84431 (9)	0.0466 (4)	
C24	−0.01820 (15)	0.75367 (16)	0.49692 (9)	0.0495 (4)	
C22	0.12647 (16)	0.55958 (17)	0.62402 (9)	0.0571 (4)	
H22	0.1513	0.4694	0.665	0.068*	
O4	−0.18158 (13)	0.74572 (15)	0.41247 (7)	0.0865 (4)	
C33	0.54160 (15)	0.64120 (16)	0.80149 (8)	0.0473 (4)	
C26	0.16313 (16)	0.76359 (16)	0.56908 (9)	0.0532 (4)	
H26	0.2123	0.8115	0.5729	0.064*	
C25	0.05410 (16)	0.82557 (16)	0.50160 (9)	0.0550 (4)	
H25	0.0295	0.9149	0.4597	0.066*	
C8	−0.06809 (15)	0.81009 (15)	0.16531 (9)	0.0520 (4)	
C7	0.30787 (16)	0.34361 (17)	0.37839 (9)	0.0569 (4)	
H7	0.2716	0.3222	0.3367	0.068*	
C3	0.30811 (15)	0.51500 (17)	0.43730 (9)	0.0557 (4)	
H3	0.2742	0.61	0.4344	0.067*	
C34	0.47117 (17)	0.79007 (16)	0.76366 (9)	0.0542 (4)	
H34	0.382	0.8372	0.7309	0.065*	
C5	0.46458 (16)	0.26540 (17)	0.50650 (10)	0.0563 (4)	
C14	−0.01841 (15)	0.98197 (17)	0.20603 (10)	0.0526 (4)	
C38	0.67299 (16)	0.57297 (17)	0.85254 (9)	0.0565 (4)	
H38	0.7203	0.4733	0.8798	0.068*	
O3	−0.17919 (15)	0.94622 (16)	0.38125 (9)	0.0982 (4)	
N3	0.57624 (17)	0.14956 (19)	0.57804 (11)	0.0811 (5)	
C23	0.01649 (16)	0.62102 (18)	0.55673 (9)	0.0575 (4)	
H23	−0.0329	0.5735	0.5521	0.069*	
C4	0.41121 (16)	0.40499 (18)	0.50469 (10)	0.0595 (4)	
H4	0.4445	0.4247	0.5486	0.071*	
C28	0.49956 (15)	0.38849 (17)	0.92809 (9)	0.0564 (4)	
H28	0.4457	0.4657	0.9472	0.068*	
C15	0.05885 (17)	1.00997 (19)	0.25729 (10)	0.0625 (4)	
H15	0.1297	0.9343	0.3021	0.075*	
C37	0.73250 (18)	0.6538 (2)	0.86244 (11)	0.0686 (5)	
H37	0.8207	0.6077	0.8962	0.082*	
C6	0.41473 (17)	0.23299 (17)	0.44433 (10)	0.0607 (4)	
H6	0.4522	0.1379	0.4464	0.073*	
C35	0.53307 (19)	0.86828 (18)	0.77451 (10)	0.0659 (4)	
H35	0.4858	0.9682	0.7484	0.079*	
C32	0.60992 (16)	0.29899 (18)	0.81561 (10)	0.0569 (4)	
H32	0.6309	0.3155	0.7587	0.068*	
C31	0.65662 (17)	0.16031 (18)	0.87176 (12)	0.0688 (5)	
H31	0.7096	0.0827	0.8528	0.083*	
C12	−0.25571 (18)	0.76574 (19)	0.13928 (11)	0.0705 (5)	
H12	−0.3363	0.7609	0.1554	0.085*	
C29	0.54716 (17)	0.2492 (2)	0.98368 (10)	0.0678 (5)	
H29	0.5259	0.2324	1.0406	0.081*	
C13	−0.18622 (17)	0.80211 (17)	0.18977 (10)	0.0611 (4)	
H13	−0.2195	0.8211	0.2401	0.073*	
C9	−0.01978 (17)	0.78373 (18)	0.09056 (10)	0.0669 (5)	
H9	0.0593	0.7909	0.0733	0.08*	
O1	0.60017 (18)	0.17750 (16)	0.64075 (10)	0.1282 (6)	
C18	−0.1473 (2)	1.2352 (2)	0.12438 (13)	0.0797 (5)	
H18	−0.2168	1.3114	0.0791	0.096*	
C19	−0.12189 (17)	1.09637 (18)	0.13879 (11)	0.0664 (4)	
H19	−0.1743	1.0796	0.1034	0.08*	
C30	0.62518 (18)	0.13607 (19)	0.95579 (12)	0.0718 (5)	
H30	0.6573	0.0421	0.9937	0.086*	
C36	0.6644 (2)	0.8003 (2)	0.82365 (12)	0.0739 (5)	
H36	0.7061	0.8537	0.8303	0.089*	
C16	0.0311 (2)	1.1497 (2)	0.24218 (13)	0.0757 (5)	
H16	0.0825	1.1675	0.2775	0.091*	
O2	0.64046 (17)	0.02998 (17)	0.57238 (10)	0.1191 (6)	
C10	−0.0895 (2)	0.7465 (2)	0.04114 (11)	0.0798 (5)	
H10	−0.0568	0.7279	−0.0093	0.096*	
C17	−0.0716 (2)	1.2626 (2)	0.17568 (14)	0.0809 (5)	
H17	−0.0895	1.3565	0.1656	0.097*	
C11	−0.2058 (2)	0.73704 (19)	0.06594 (12)	0.0763 (5)	
H11	−0.2516	0.7108	0.0327	0.092*	

### Atomic displacement parameters (Å^2^)

**Table:**

	*U*^11^	*U*^22^	*U*^33^	*U*^12^	*U*^13^	*U*^23^
N5	0.0556 (8)	0.0518 (8)	0.0430 (7)	−0.0262 (7)	−0.0071 (6)	−0.0092 (6)
N4	0.0652 (8)	0.0481 (8)	0.0504 (7)	−0.0299 (7)	−0.0176 (6)	−0.0023 (6)
N2	0.0429 (7)	0.0548 (9)	0.0509 (7)	−0.0187 (7)	−0.0022 (6)	−0.0142 (6)
N1	0.0501 (8)	0.0528 (8)	0.0600 (8)	−0.0181 (7)	−0.0122 (6)	−0.0144 (7)
N6	0.0588 (9)	0.0766 (11)	0.0497 (8)	−0.0272 (9)	−0.0037 (7)	−0.0193 (8)
C1	0.0475 (9)	0.0592 (11)	0.0478 (9)	−0.0247 (8)	0.0018 (7)	−0.0195 (8)
C21	0.0536 (9)	0.0470 (9)	0.0411 (8)	−0.0247 (8)	0.0008 (7)	−0.0129 (7)
C2	0.0406 (8)	0.0528 (10)	0.0449 (8)	−0.0219 (8)	0.0053 (7)	−0.0151 (7)
C20	0.0615 (10)	0.0488 (9)	0.0443 (8)	−0.0285 (8)	−0.0058 (7)	−0.0056 (7)
C27	0.0457 (8)	0.0468 (9)	0.0421 (8)	−0.0218 (7)	−0.0053 (7)	−0.0090 (7)
C24	0.0472 (9)	0.0550 (10)	0.0402 (8)	−0.0195 (8)	−0.0019 (7)	−0.0170 (8)
C22	0.0676 (10)	0.0580 (10)	0.0466 (9)	−0.0378 (9)	−0.0011 (8)	−0.0068 (8)
O4	0.0908 (9)	0.1161 (11)	0.0650 (7)	−0.0651 (9)	−0.0125 (6)	−0.0210 (7)
C33	0.0509 (9)	0.0502 (9)	0.0422 (8)	−0.0259 (8)	0.0020 (7)	−0.0151 (7)
C26	0.0630 (10)	0.0475 (9)	0.0506 (9)	−0.0292 (8)	−0.0043 (8)	−0.0140 (8)
C25	0.0665 (10)	0.0428 (9)	0.0475 (9)	−0.0212 (8)	−0.0045 (8)	−0.0130 (7)
C8	0.0478 (9)	0.0496 (9)	0.0476 (9)	−0.0182 (8)	−0.0061 (7)	−0.0106 (7)
C7	0.0664 (10)	0.0591 (11)	0.0465 (9)	−0.0314 (9)	0.0041 (8)	−0.0183 (8)
C3	0.0507 (9)	0.0525 (10)	0.0601 (10)	−0.0208 (8)	−0.0022 (8)	−0.0209 (8)
C34	0.0571 (10)	0.0516 (10)	0.0507 (9)	−0.0256 (9)	0.0046 (7)	−0.0142 (8)
C5	0.0541 (10)	0.0544 (10)	0.0526 (9)	−0.0277 (9)	−0.0044 (7)	−0.0055 (8)
C14	0.0430 (9)	0.0544 (10)	0.0580 (9)	−0.0202 (8)	0.0097 (7)	−0.0217 (8)
C38	0.0546 (10)	0.0560 (10)	0.0563 (9)	−0.0236 (8)	−0.0023 (8)	−0.0206 (8)
O3	0.0915 (10)	0.0763 (10)	0.0864 (9)	−0.0217 (8)	−0.0377 (8)	−0.0048 (8)
N3	0.0853 (12)	0.0663 (12)	0.0739 (11)	−0.0364 (10)	−0.0212 (9)	−0.0002 (9)
C23	0.0615 (10)	0.0703 (11)	0.0501 (9)	−0.0422 (9)	0.0017 (8)	−0.0155 (9)
C4	0.0587 (10)	0.0658 (12)	0.0561 (9)	−0.0324 (9)	−0.0049 (8)	−0.0191 (9)
C28	0.0526 (9)	0.0550 (10)	0.0468 (9)	−0.0148 (8)	−0.0024 (7)	−0.0168 (8)
C15	0.0593 (10)	0.0665 (12)	0.0648 (10)	−0.0310 (9)	0.0082 (8)	−0.0257 (9)
C37	0.0586 (10)	0.0853 (14)	0.0743 (11)	−0.0373 (11)	0.0047 (9)	−0.0389 (11)
C6	0.0704 (11)	0.0482 (10)	0.0559 (9)	−0.0256 (9)	0.0012 (9)	−0.0128 (8)
C35	0.0798 (13)	0.0569 (11)	0.0693 (11)	−0.0374 (10)	0.0163 (10)	−0.0254 (9)
C32	0.0579 (10)	0.0623 (11)	0.0560 (9)	−0.0320 (9)	0.0119 (8)	−0.0226 (9)
C31	0.0585 (10)	0.0509 (11)	0.0900 (13)	−0.0186 (9)	0.0073 (9)	−0.0280 (10)
C12	0.0676 (11)	0.0750 (12)	0.0608 (11)	−0.0430 (10)	−0.0103 (9)	0.0020 (9)
C29	0.0643 (11)	0.0710 (13)	0.0465 (9)	−0.0261 (10)	−0.0008 (8)	−0.0032 (9)
C13	0.0571 (10)	0.0698 (11)	0.0460 (9)	−0.0299 (9)	−0.0028 (8)	−0.0074 (8)
C9	0.0501 (9)	0.0771 (12)	0.0603 (10)	−0.0206 (9)	0.0024 (8)	−0.0233 (9)
O1	0.1500 (15)	0.0965 (11)	0.1025 (11)	−0.0444 (11)	−0.0683 (10)	−0.0096 (9)
C18	0.0667 (12)	0.0581 (12)	0.0895 (13)	−0.0163 (10)	0.0093 (10)	−0.0166 (11)
C19	0.0527 (10)	0.0562 (11)	0.0771 (11)	−0.0188 (9)	−0.0012 (9)	−0.0179 (9)
C30	0.0653 (11)	0.0499 (11)	0.0726 (12)	−0.0215 (10)	−0.0104 (9)	0.0048 (9)
C36	0.0821 (14)	0.0855 (15)	0.0879 (12)	−0.0570 (12)	0.0223 (11)	−0.0460 (11)
C16	0.0828 (14)	0.0824 (14)	0.0848 (13)	−0.0501 (12)	0.0265 (11)	−0.0419 (12)
O2	0.1206 (13)	0.0599 (9)	0.1231 (12)	−0.0158 (9)	−0.0382 (10)	−0.0062 (9)
C10	0.0748 (13)	0.0883 (14)	0.0648 (11)	−0.0240 (12)	−0.0008 (10)	−0.0364 (10)
C17	0.0874 (14)	0.0638 (13)	0.0968 (15)	−0.0358 (12)	0.0329 (12)	−0.0368 (12)
C11	0.0857 (14)	0.0690 (12)	0.0677 (12)	−0.0353 (11)	−0.0172 (10)	−0.0174 (10)

### Geometric parameters (Å, º)

**Table:**

N5—C20	1.2774 (17)	C14—C15	1.386 (2)
N5—N4	1.3632 (15)	C14—C19	1.386 (2)
N4—C33	1.4062 (17)	C38—C37	1.376 (2)
N4—C27	1.4335 (17)	C38—H38	0.93
N2—C1	1.2847 (18)	N3—O2	1.2142 (19)
N2—N1	1.3633 (16)	N3—O1	1.2154 (18)
N1—C14	1.4021 (19)	C23—H23	0.93
N1—C8	1.4368 (18)	C4—H4	0.93
N6—O3	1.2192 (17)	C28—C29	1.376 (2)
N6—O4	1.2217 (16)	C28—H28	0.93
N6—C24	1.4605 (19)	C15—C16	1.380 (2)
C1—C2	1.4564 (19)	C15—H15	0.93
C1—H1	0.93	C37—C36	1.365 (2)
C21—C22	1.3856 (19)	C37—H37	0.93
C21—C26	1.3966 (19)	C6—H6	0.93
C21—C20	1.4538 (19)	C35—C36	1.378 (2)
C2—C7	1.388 (2)	C35—H35	0.93
C2—C3	1.3914 (19)	C32—C31	1.375 (2)
C20—H20	0.93	C32—H32	0.93
C27—C28	1.3738 (19)	C31—C30	1.374 (2)
C27—C32	1.377 (2)	C31—H31	0.93
C24—C23	1.370 (2)	C12—C11	1.363 (2)
C24—C25	1.3762 (19)	C12—C13	1.384 (2)
C22—C23	1.380 (2)	C12—H12	0.93
C22—H22	0.93	C29—C30	1.361 (2)
C33—C34	1.3843 (19)	C29—H29	0.93
C33—C38	1.3917 (19)	C13—H13	0.93
C26—C25	1.3745 (19)	C9—C10	1.381 (2)
C26—H26	0.93	C9—H9	0.93
C25—H25	0.93	C18—C17	1.369 (2)
C8—C13	1.371 (2)	C18—C19	1.381 (2)
C8—C9	1.372 (2)	C18—H18	0.93
C7—C6	1.379 (2)	C19—H19	0.93
C7—H7	0.93	C30—H30	0.93
C3—C4	1.372 (2)	C36—H36	0.93
C3—H3	0.93	C16—C17	1.372 (2)
C34—C35	1.375 (2)	C16—H16	0.93
C34—H34	0.93	C10—C11	1.361 (2)
C5—C6	1.366 (2)	C10—H10	0.93
C5—C4	1.373 (2)	C17—H17	0.93
C5—N3	1.464 (2)	C11—H11	0.93
			
C20—N5—N4	119.59 (12)	O1—N3—C5	118.34 (17)
N5—N4—C33	116.35 (11)	C24—C23—C22	118.37 (14)
N5—N4—C27	122.46 (11)	C24—C23—H23	120.8
C33—N4—C27	121.17 (11)	C22—C23—H23	120.8
C1—N2—N1	120.25 (12)	C3—C4—C5	119.16 (14)
N2—N1—C14	117.33 (12)	C3—C4—H4	120.4
N2—N1—C8	120.82 (12)	C5—C4—H4	120.4
C14—N1—C8	121.85 (12)	C27—C28—C29	119.80 (15)
O3—N6—O4	123.20 (14)	C27—C28—H28	120.1
O3—N6—C24	118.16 (15)	C29—C28—H28	120.1
O4—N6—C24	118.63 (15)	C16—C15—C14	120.27 (16)
N2—C1—C2	119.80 (13)	C16—C15—H15	119.9
N2—C1—H1	120.1	C14—C15—H15	119.9
C2—C1—H1	120.1	C36—C37—C38	121.16 (16)
C22—C21—C26	118.45 (13)	C36—C37—H37	119.4
C22—C21—C20	118.98 (13)	C38—C37—H37	119.4
C26—C21—C20	122.57 (13)	C5—C6—C7	118.90 (14)
C7—C2—C3	118.23 (13)	C5—C6—H6	120.6
C7—C2—C1	119.76 (13)	C7—C6—H6	120.6
C3—C2—C1	122.00 (13)	C34—C35—C36	120.75 (16)
N5—C20—C21	120.75 (13)	C34—C35—H35	119.6
N5—C20—H20	119.6	C36—C35—H35	119.6
C21—C20—H20	119.6	C31—C32—C27	119.51 (14)
C28—C27—C32	120.13 (14)	C31—C32—H32	120.2
C28—C27—N4	119.59 (14)	C27—C32—H32	120.2
C32—C27—N4	120.28 (13)	C30—C31—C32	120.17 (16)
C23—C24—C25	122.04 (13)	C30—C31—H31	119.9
C23—C24—N6	118.78 (14)	C32—C31—H31	119.9
C25—C24—N6	119.18 (14)	C11—C12—C13	119.87 (16)
C23—C22—C21	121.46 (14)	C11—C12—H12	120.1
C23—C22—H22	119.3	C13—C12—H12	120.1
C21—C22—H22	119.3	C30—C29—C28	120.25 (15)
C34—C33—C38	119.20 (13)	C30—C29—H29	119.9
C34—C33—N4	120.96 (12)	C28—C29—H29	119.9
C38—C33—N4	119.82 (13)	C8—C13—C12	119.74 (15)
C25—C26—C21	120.58 (14)	C8—C13—H13	120.1
C25—C26—H26	119.7	C12—C13—H13	120.1
C21—C26—H26	119.7	C8—C9—C10	119.48 (16)
C26—C25—C24	119.09 (14)	C8—C9—H9	120.3
C26—C25—H25	120.5	C10—C9—H9	120.3
C24—C25—H25	120.5	C17—C18—C19	121.06 (18)
C13—C8—C9	120.19 (14)	C17—C18—H18	119.5
C13—C8—N1	119.42 (14)	C19—C18—H18	119.5
C9—C8—N1	120.35 (14)	C18—C19—C14	120.02 (17)
C6—C7—C2	121.11 (14)	C18—C19—H19	120
C6—C7—H7	119.4	C14—C19—H19	120
C2—C7—H7	119.4	C29—C30—C31	120.14 (16)
C4—C3—C2	120.89 (14)	C29—C30—H30	119.9
C4—C3—H3	119.6	C31—C30—H30	119.9
C2—C3—H3	119.6	C37—C36—C35	119.25 (16)
C35—C34—C33	119.97 (14)	C37—C36—H36	120.4
C35—C34—H34	120	C35—C36—H36	120.4
C33—C34—H34	120	C17—C16—C15	120.79 (18)
C6—C5—C4	121.65 (14)	C17—C16—H16	119.6
C6—C5—N3	119.90 (15)	C15—C16—H16	119.6
C4—C5—N3	118.45 (15)	C11—C10—C9	120.34 (17)
C15—C14—C19	118.75 (15)	C11—C10—H10	119.8
C15—C14—N1	121.37 (14)	C9—C10—H10	119.8
C19—C14—N1	119.87 (14)	C18—C17—C16	119.10 (18)
C37—C38—C33	119.64 (15)	C18—C17—H17	120.4
C37—C38—H38	120.2	C16—C17—H17	120.4
C33—C38—H38	120.2	C10—C11—C12	120.37 (17)
O2—N3—O1	122.90 (17)	C10—C11—H11	119.8
O2—N3—C5	118.76 (17)	C12—C11—H11	119.8
			
C20—N5—N4—C33	−174.28 (13)	C34—C33—C38—C37	−1.9 (2)
C20—N5—N4—C27	4.3 (2)	N4—C33—C38—C37	176.39 (13)
C1—N2—N1—C14	−175.43 (13)	C6—C5—N3—O2	13.3 (2)
C1—N2—N1—C8	4.2 (2)	C4—C5—N3—O2	−167.54 (17)
N1—N2—C1—C2	178.39 (11)	C6—C5—N3—O1	−166.41 (17)
N2—C1—C2—C7	−169.14 (13)	C4—C5—N3—O1	12.7 (2)
N2—C1—C2—C3	10.4 (2)	C25—C24—C23—C22	0.8 (2)
N4—N5—C20—C21	179.12 (12)	N6—C24—C23—C22	−179.75 (13)
C22—C21—C20—N5	−175.58 (14)	C21—C22—C23—C24	0.2 (2)
C26—C21—C20—N5	4.5 (2)	C2—C3—C4—C5	2.6 (2)
N5—N4—C27—C28	−108.19 (16)	C6—C5—C4—C3	−1.9 (2)
C33—N4—C27—C28	70.35 (18)	N3—C5—C4—C3	178.97 (13)
N5—N4—C27—C32	71.90 (19)	C32—C27—C28—C29	0.9 (2)
C33—N4—C27—C32	−109.55 (15)	N4—C27—C28—C29	−178.97 (13)
O3—N6—C24—C23	168.64 (15)	C19—C14—C15—C16	1.1 (2)
O4—N6—C24—C23	−12.6 (2)	N1—C14—C15—C16	−179.74 (14)
O3—N6—C24—C25	−11.9 (2)	C33—C38—C37—C36	0.6 (2)
O4—N6—C24—C25	166.90 (14)	C4—C5—C6—C7	−0.2 (2)
C26—C21—C22—C23	−0.9 (2)	N3—C5—C6—C7	178.90 (13)
C20—C21—C22—C23	179.14 (14)	C2—C7—C6—C5	1.7 (2)
N5—N4—C33—C34	17.35 (19)	C33—C34—C35—C36	−0.8 (2)
C27—N4—C33—C34	−161.29 (13)	C28—C27—C32—C31	−0.8 (2)
N5—N4—C33—C38	−160.95 (12)	N4—C27—C32—C31	179.12 (13)
C27—N4—C33—C38	20.4 (2)	C27—C32—C31—C30	0.2 (2)
C22—C21—C26—C25	0.7 (2)	C27—C28—C29—C30	−0.5 (2)
C20—C21—C26—C25	−179.39 (14)	C9—C8—C13—C12	0.7 (2)
C21—C26—C25—C24	0.3 (2)	N1—C8—C13—C12	−177.01 (13)
C23—C24—C25—C26	−1.0 (2)	C11—C12—C13—C8	0.5 (2)
N6—C24—C25—C26	179.51 (13)	C13—C8—C9—C10	−1.2 (2)
N2—N1—C8—C13	86.15 (18)	N1—C8—C9—C10	176.53 (14)
C14—N1—C8—C13	−94.20 (17)	C17—C18—C19—C14	0.0 (3)
N2—N1—C8—C9	−91.60 (17)	C15—C14—C19—C18	−0.6 (2)
C14—N1—C8—C9	88.05 (18)	N1—C14—C19—C18	−179.78 (14)
C3—C2—C7—C6	−1.1 (2)	C28—C29—C30—C31	−0.1 (3)
C1—C2—C7—C6	178.45 (13)	C32—C31—C30—C29	0.3 (2)
C7—C2—C3—C4	−1.1 (2)	C38—C37—C36—C35	0.7 (2)
C1—C2—C3—C4	179.40 (13)	C34—C35—C36—C37	−0.6 (2)
C38—C33—C34—C35	2.0 (2)	C14—C15—C16—C17	−1.0 (3)
N4—C33—C34—C35	−176.28 (13)	C8—C9—C10—C11	0.5 (3)
N2—N1—C14—C15	2.2 (2)	C19—C18—C17—C16	0.1 (3)
C8—N1—C14—C15	−177.42 (13)	C15—C16—C17—C18	0.4 (3)
N2—N1—C14—C19	−178.56 (12)	C9—C10—C11—C12	0.8 (3)
C8—N1—C14—C19	1.8 (2)	C13—C12—C11—C10	−1.2 (3)

### Hydrogen-bond geometry (Å, º)

*Cg*1, *Cg*2 and *Cg*3 are the centroids of the C21–C26, C33–C38 and C8–C13 rings, respectively.

**Table:**

*D*—H···*A*	*D*—H	H···*A*	*D*···*A*	*D*—H···*A*
C3—H3···*Cg*1^i^	0.93	2.92	3.4080 (18)	114
C29—H29···*Cg*2^ii^	0.93	2.80	3.6875 (18)	161
C7—H7···*Cg*2	0.93	2.83	3.4223 (16)	123
C30—H30···*Cg*3^iii^	0.93	2.84	3.698 (2)	154
C6—H6···O2^iv^	0.93	2.60	3.342 (3)	138
C15—H15···N2	0.93	2.43	2.750 (2)	100

Symmetry codes: (i) −*x*+1, −*y*+1, −*z*+1; (ii) −*x*+1, −*y*+1, −*z*; (iii) −*x*, −*y*+2, −*z*; (iv) −*x*+1, −*y*, −*z*+1.
